# Spliceosomal introns in the 5′ untranslated region of plant BTL RING-H2 ubiquitin ligases are evolutionary conserved and required for gene expression

**DOI:** 10.1186/1471-2229-13-179

**Published:** 2013-11-14

**Authors:** Victor Aguilar-Hernández, Plinio Guzmán

**Affiliations:** 1 Departamento de Ingeniería Genética, Centro de Investigación y de Estudios Avanzados, Unidad Irapuato, Apartado Postal 629, Irapuato, Gto 36821, Mexico

**Keywords:** 5′-UTR intron, IMEter score, Ubiquitin ligases, Intron-mediated enhancement, MHX, Polyubiquitin genes

## Abstract

**Background:**

Introns located close to the 5′ end of a gene or in the 5′ untranslated region often exert positive effects on gene expression. This effect, known as intron-mediated enhancement (IME), has been observed in diverse eukaryotic organisms, including plants. The sequences involved in IME seem to be spread across the intron and function in an additive manner. The IMEter algorithm was developed to predict plant introns that may enhance gene expression. We have identified several plant members of the BTL class of E3s, which may have orthologs across eukaryotes, that contain a 5′UTR intron. The RING finger E3 ligases are key enzymes of the ubiquitination system that mediate the transfer of ubiquitin to substrates.

**Results:**

In this study, we retrieved *BTL* sequences from several angiosperm species and found that 5′UTR introns showing a strong IMEter score were predicted, suggesting that they may be conserved by lineage. Promoter-GUS fusion lines were used to confirm the IME effect of these 5′UTR introns on gene expression. IMEter scores of *BTLs* were compared with the 5′UTR introns of two gene families *MHX* and polyubiquitin genes.

**Conclusions:**

Analysis performed in two Arabidopsis *BTL* E3 ligases genes indicated that the 5′UTR introns were essential for gene expression in all the tissues tested. Comparison of the average 5′UTR intron size on three gene families in ten angiosperm species suggests that a prevalent size for a 5′UTR intron is in the range of 600 nucleotides, and that the overall IMEter score within a gene family is preserved across several angiosperms. Our results indicated that gene expression dependent on a 5′UTR intron is an efficient regulatory mechanism in BTL E3 ligases that has been preserved throughout plant evolution.

## Background

Spliceosomal introns are evolutionary conserved features ubiquitously found in eukaryotic genomes that interrupt the coding sequences of genes. Introns are transcribed into pre-mRNA, which is then processed to produce mature mRNA. The spliceosome is a conserved and versatile ribonucleoprotein machinery that generates the mature mRNA by splicing the exons via precise excision of the introns at the donor and acceptor sites [1,2]. Alternative splicing of introns enhances proteome diversity as well as serves as a mechanism for regulating gene expression. Over 40% of plant genes (*Arabidopsis thaliana* and *Oryza sativa*) and over 90% of human genes are processed by alternative splicing to generate multiple and assorted mature mRNA molecules [3-6].

Recent studies have identified specific introns that are able to enhance gene expression. In some cases, these introns enhance mRNA accumulation 10-fold higher than normal levels. This effect, known as intron-mediated enhancement (IME), is likely to be a conserved mechanism throughout evolution, as it has been described in diverse plant, animal and fungal species [7-10]. The mechanisms underlying IME have not been clearly established, but a mechanism distinct from that of conventional transcriptional enhancer elements has been envisioned. Introns involved in IME should be located within the transcription unit in the sense orientation and close to the 5′ terminal end of the gene. IME progressively declines as the intron is located farther from the 5′ end, and is inactive when the intron is located at the 3′ untranslated region. Sequences consisting of redundant motifs that are dispersed throughout the intron are shown to be required for IME. These sequences are frequently located toward the 5′ end of the intron and exert additive effects on expression [8,11,12].

Spliceosomal introns are also present in the untranslated regions (UTRs) of genes. These 5′UTR introns are generally longer than introns within coding sequences and may influence gene expression, mRNA stability or mRNA export. Conversely, introns located in the 3′UTR are capable of downregulating levels of gene expression [13,14]. IME has been observed in several plant introns, usually prompted by introns located in the 5′UTR. The extent of IME depends on intron sequence composition and position and on the spatiotemporal expression features of the promoter. The enhancement of expression from 2- to 10-fold or more can be expected, and a stronger effect has been observed in monocots compared to eudicots [12,15,16].

The specific sequences in introns involved in IME are not restricted to discrete motifs. On the contrary, the sequences seem to be scattered throughout the intron and function in an additive fashion [17]. Although these sequences have not yet been identified, an algorithm to analyze introns that may stimulate expression in plants has been developed. This algorithm, known as the IMEter is based on word pattern frequency at introns. The IMEter score reveals the similarity between an input intron to introns located proximal to the start of transcription or to distal introns. IMEter gives high scores to sequences that resemble proximal introns and low scores to sequences that resemble distal introns [17,18].

The *BCA2* zinc finger A*TL* (BTL) family of RING-H2 finger E3 ligases in plants are possible orthologs of the mammalian Rabring7/BCA2 RING-H2 E3 ligases [19,20]; BTLs share some common structural features with the *A*rabidopsis *T*óxicos en *L*evadura (ATL) family of RING finger ligases [21]. E3 ligases are components of the ubiquitin proteasome system (UPS) that recognize specific substrates for degradation and mediates the transfer of the ubiquitin [22]. Although functional analysis of *Rabring7*/*BCA2*/*BTLs* is scarce, this class of E3s has been identified across eukaryotes, in plants, animals, fungi, and protozoa genomes, and has shown gene expansion in land plants [20]. The best characterized member of this class in humans is Rab7-interacting RING finger protein (Rabring7), also known as Breast Cancer Associated gene 2 (BCA2), which is involved in intracellular vesicle traffic regulation and was also identified as a differentially expressed gene in cancerous mammary epithelial cell lines [23,24]. Few putative orthologs have been identified thus far in plants, including CIP8, which supports the function of the E3 ligase COP1 in ubiquitination; AtRDUF1 and AtRZF1 that may function in the response to dehydration and drought, and *RHC1* which is involved in root architecture [25-28].

**Figure 1:**
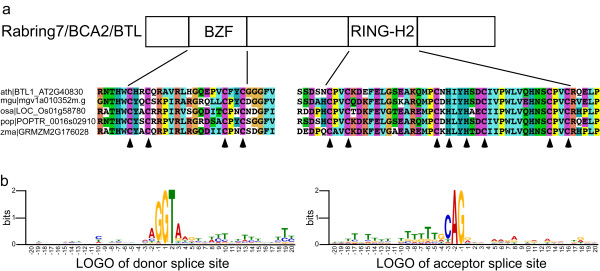
**General features of *BTLs*. a**. A schematic representation of a canonical Rabring7/BCA2/BTL E3 ligase showing the relative position of the BZF and RING-H2 domains. A sequence LOGO comparison between the two RING-H2 domains is shown; LOGOs were generated from the collected Rabring7/BCA2/BTLs sequences and from a previous analysis of the E3 ATLs. An alignment of five representative BTLs with the BZF and the RING-H2 regions displayed below. ClustalX was used for sequence alignment and a default color code was applied. The arrowheads point to the residues involved in zinc ligation in the RING-H2 domain and the conserved cysteines in the BZF domain. **b**. Donor and acceptor sites of 5′UTR introns from 63 *BTLs*. Twenty nucleotides of exon and intron sequences at the donor site were used to obtain the LOGO, as described in Methods.

The Rabring7/BCA2/BTLs E3 ligases contain a RING-H2 domain and a C2/C2 zinc finger, and share at least two common sequence LOGOs located between the two zinc fingers (see Figure 1a) [19,20]. Analysis of gene architecture revealed two to nine spliceosomal introns within the coding sequence in most animal, fungi and protist *Rabring7*/*BCA2*/*BTL* genes. Although there are no introns within coding sequences of *BTL* genes, they were predicted in the 5′UTR of several genes. Specifically, in *A. thaliana* and *O. sativa*, eight and nine out of the seventeen *BTL* genes, respectively, contained an intron in the 5′UTR [20].

In this report, we evaluated the conservation of BTL 5′UTR introns in fourteen angiosperm species. We confirmed that 5′UTR introns are conserved by lineage, with most showing a strong IMEter score. Analysis of the effect of the 5′UTR intron on the expression of two *AthBTL* genes showed that these introns are required for gene expression.

## Results and discussion

### Identification of BTLs in 14 angiosperm species

In a previous work, we reported that 5′UTR spliceosomal introns were commonly found in BTLs, specifically as identified from *A. thaliana* and *O. sativa* cDNA clones. The BTLs were classified in six groups based on phylogenetic analysis, and two of these groups, denoted as A and B, contained mostly BTLs with 5′UTR intron (see Table 1). These two phylogenetic delimited groups showed different domain structure as assessed by sequence LOGO composition, suggesting that each one of them function with distinct substrates [20]. To evaluate the significance of the 5′UTR spliceosomal introns in the BTL genes, we examined their conservation in fourteen angiosperm species, which included ten eudicots and four monocots (Table 1). These fourteen species included BTLs from both groups A and B with annotated 5′UTR introns, except for *Aquilegia caerulea* that only contains one BTL member in group A. In 62 out of the 73 *BTL* genes, the intron was annotated (Table 1). This data suggests that 5′UTR spliceosomal introns were acquired early in the angiosperm lineage, and that this is a conserved feature in these two BTL groups.

**Table 1:** **Retrieved *BTL *** **genes in groups A and B from 14 angiosperm genomes containing a 5′UTR intron**

	**Species**	**Abbreviation**	**Total BTLs**	Group A*	Group A**	Group B*	Group B**
				**Total**	**5′UTR-I**	**Total**	**5′UTR-I**
Monocots	*Oryza sativa* japonica	osa	17	3	2	4	4
	*Brachypodium distachyon*	bdi	15	2	2	4	2
	*Zea mays*	zma	20	3	2	6	5
	*Sorghum bicolor*	sbi	13	1	1	4	4
Eudicots	*Aquilegia caerulea*	aco	7	1	1	-	-
	*Mimulus guttatus*	mgu	10	2	1	1	1
	*Vitis vinifera*	vvi	7	1	1	1	1
	*Eucalyptus grandis*	egr	15	3	3	3	2
	*Citrus sinensis*	csi	10	1	1	3	1
	*Arabidopsis thaliana*	ath	17	3	3	4	4
	*Capsella rubella*	cru	17	3	3	4	4
	*Thellungiella halophila*	tha	16	3	2	4	3
	*Cucumis sativus*	cat	10	2	2	1	1
	*Populus trichocarpa*	pop	21	2	2	4	4

*Total number of BTLs in the group.

** Number of BTLs in the group containing a 5′UTR intron.

To confirm spliceosomal intron annotations in *BTLs*, sequence LOGOs were generated over the nucleotides at the splicing donor and acceptor sites (Figure 1b). Canonical sequences were observed at the generated LOGOs, with GT at the donor site and AG at the acceptor site, indicating that annotated introns from *A. thaliana* and *O. sativa* as well as from the selected plant genomes are likely to be valid spliceosomal introns [29]. Moreover, there was no evidence of additional conserved sequences within 40 nucleotides in close proximity to the donor or acceptor sites (Figure 1b).

### IMEter scores of 5′UTR introns of group A and B BTL genes

**Figure 2:**
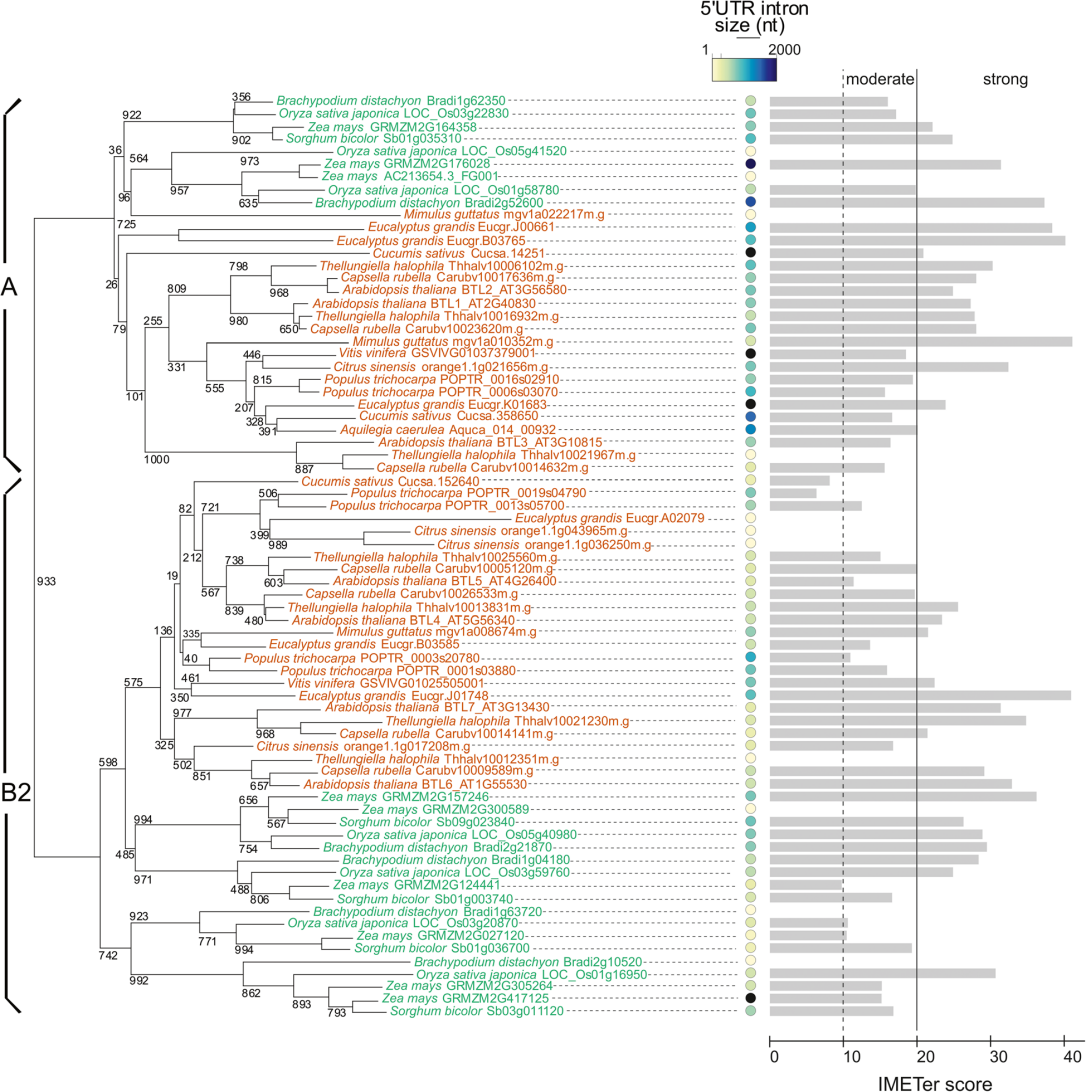
**IMEter score and intron size of BTL genes containing a 5′UTR-intron.** A phylogenetic tree of the 81 retrieved BTL sequences is display; it was generated as described in Methods. The bootstrap values of 1000 replicates are shown at the branches. The intron size scale goes from 1 to 2000 nucleotides. Introns longer than 2000 nucleotides have the maximum value (zma|GRMZM2G417125, 3919 nt; zma|GRMZM2G176028, 6571 nt; cat|Cucsa.14251, 2149 nt; vvi|GSVIVG01037379001, 3518 nt, and egr|Eucgr.K01683, 3411 nt). The average size of introns in groups A and B was calculated after eliminating the extreme values, resulting in 1,133+/-801 nucleotides for group A and 578+/-444 for group B; the average sizes in monocots and eudicots were similar when measured independently. IMEter v2.0 scores: moderate enhancement, between 10 and 20, and strong enhancement, more than 20.

A phylogenetic tree of BTLs from groups A and B from the selected species resolved two major clades, each one grouping the corresponding sequences. Inspection of intron size revealed that in each group, an average intron size was relatively preserved. In group A, introns had an average size of 1,133+/-801 nucleotides, and in group B, introns had an average size of 578+/-444 nucleotides (Figure 2). This difference suggests that intron size is a trait, and that it was likely established before the split between monocots and eudicots.

As described for several plant 5′UTR introns, we speculated that the 5′UTR introns in these BTLs might also be involved in gene expression. To evaluate this hypothesis, we used the intron IMEter score. The IMEter score determines whether an intron is more similar to proximal than distal introns, hence more likely to encode elements that enhance gene expression. Although the IMEter was developed with *A. thaliana* intron sequences, it is also effective in predictions of other plant species. The higher the score of the intron indicates a higher probability that the intron enhances expression. An IMEter score above 20 was established as predicting introns that are likely to have a strong effect on enhancing expression, while an IMEter score between 10 and 20 likely has a moderate effect on gene expression. Approximately 80% of all *A. thaliana* introns have an IMEter score less than or equal to 9.25 (see http://korflab.ucdavis.edu/cgi-bin/web-imeter2.pl). We found that among the 62 BTLs tested, 39 had a strong IMEter score, 20 showed a moderate score and 3 had a low score of less than 10 (Figure 2). This IMEter scoring predicts that the 5′UTR introns in BTLs may enhance gene expression in a moderate or strong fashion.

**Figure 3:**
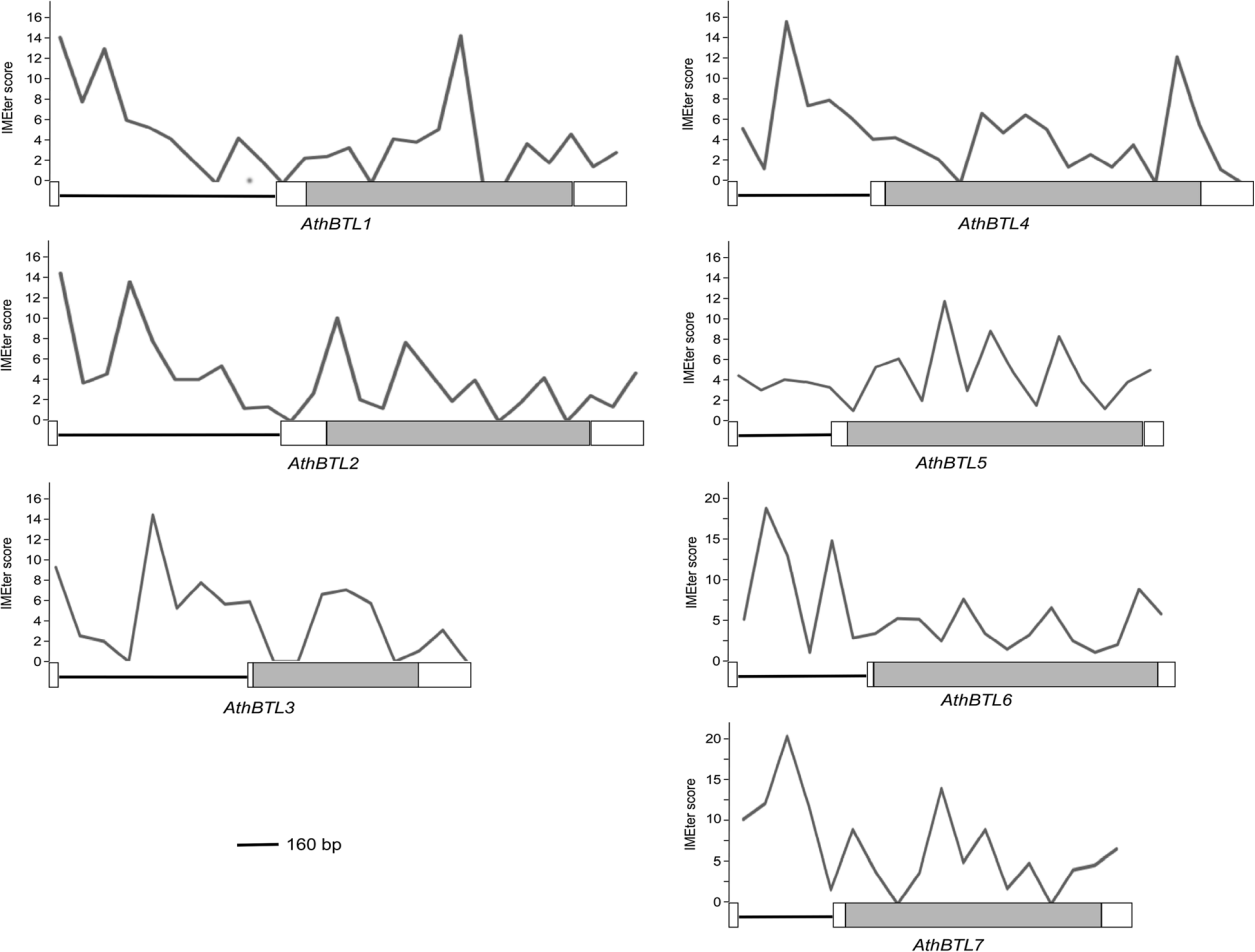
**IMEter of 5′UTR introns in *AthBTL *from groups A and B.** Distribution of IMEter scores of the seven *A. thaliana BTL* genes. In the horizontal axis, the genomic exon-intron structure is shown. The lines represent introns and boxes denote exons. The coding sequence is in a shadowed box and the 5′UTR and 3′UTR are shown in open boxes. The 5′UTR is represented by continuous line. The vertical axis shows the IMEter v2.0 score density in 80 nucleotide windows.

To provide additional information regarding the presence of sequences that enhance expression in the 5′UTR intron, the IMEter scores were determined within the gene length of the seven *A. thaliana BTL*s. Except for *AthBTL5*, which displayed a low IMEter score of the 5′UTR intron, *AthBTL1*, *AthBTL2*, *AthBTL3*, *AthBTL4*, *AthBTL6* and *AthBTL7* showed strong scores, supporting the speculation that the 5′ region of these genes contain sequences involved in the enhancement of gene expression (Figure 3).

### Effect of a 5′UTR intron on BTL gene expression in A. thaliana

**Figure 4:**
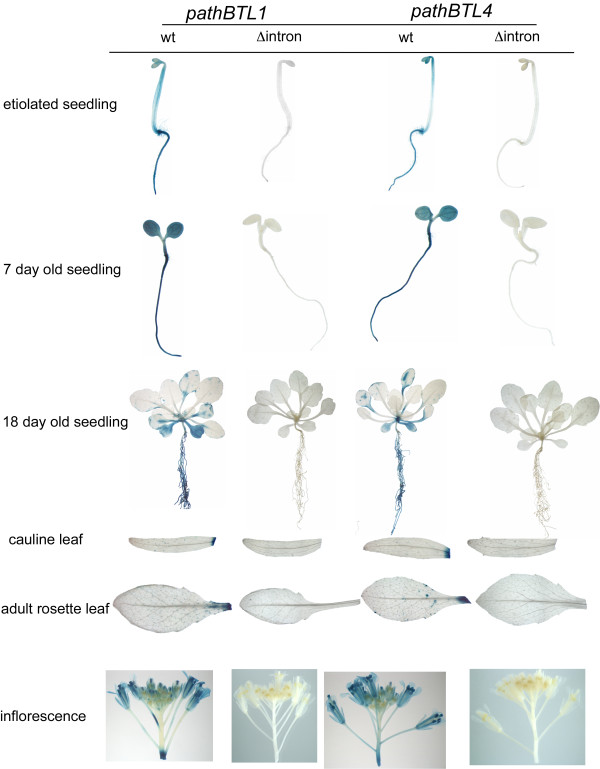
**Histochemical GUS assays of transgenic lines containing *AthBTL1 *and *AthBTL4 *promoters.** Representative samples of three-day-old dark-grown seedlings, 6- and 18-day-old light-grown seedlings, adult rosette and cauline leaves, and inflorescence from each of the transgenic lines are shown.

**Figure 5:**
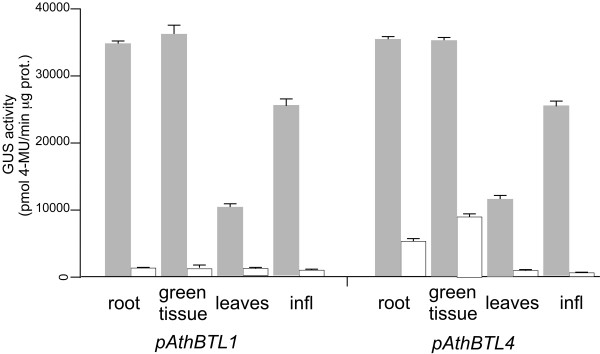
**Quantitative GUS assay of the *AthBTL1 *and *AthBTL4 *promoter lines.** The mean values and standard deviations of GUS activity were calculated from three replicates and represented as nanomoles of 4-methyl umbelliferone per milligram protein per minute, as described in Methods. The gray bars represent the expression of the p*AthBTL1*: GUS and p*AthBTL4*: GUS fusions harbouring the 5′UTR intron whereas empty bars represent the expression of the intronless promoter: GUS fusions.

To determine the effect of the 5′UTR introns of *BTLs* on gene expression, we examined promoter-GUS fusions with or without the 5′UTR intron in two *A. thaliana BTL* genes, *AthBTL1* and *AthBTL4*. Histochemical analysis was performed in several tissues and growth conditions from etiolated and young light-grown seedlings to adult tissues and inflorescences (Figure 4). The *AthBTL1* and *AthBTL4* promoters displayed GUS expression from the native promoters throughout etiolated or light-grown young seedlings. In 18-day-old seedlings, GUS expression was mainly detected in the petiole of the leaves and in the cotyledons. Expression was highly reduced throughout adult leaves, where it was detected primary in trichomes. GUS staining was readily observed in inflorescences in lines with either promoter construct (Figure 4). GUS activity was abolished in the intronless constructs of both promoters, suggesting that the 5′UTR introns in *AthBTL1* and *AthBTL4* harbor elements involved in enhancing the level of transcription. The specific GUS activity in lines carrying the wild-type promoter constructs was between 70-100 times higher in seedlings (roots and green tissue) and inflorescences compared to the intronless lines, and between 15-30 times higher in leaves where expression from these two promoters was much less intense (Figure 5).

### Comparison of the IMEter scores of 5′UTR introns from BTLs, MHX and polyubiquitin genes in 10 angiosperm species

Several 5′ UTR introns have been previously shown to enhance gene expression. Among them, the *MHX* transporter that encodes a Mg^2+^/H^+^ exchanger and the polyubiquitin genes that encode ubiquitin monomers in tandem disposition, have been the subject of several studies [30-33]. To have an appraisal of the IME effect on the BTL family, we compared IMEter scores from *BTL*, *MHX* and polyubiquitin genes. We identified 5′UTR intron sequences from three monocot and seven eudicot species. A single *MHX* 5′UTR intron sequence was retrieved from each species, whereas one to six intron sequences from polyubiquitin genes (see Additional file 1). The position of the intron in polyubiquitin genes is highly conserved, located just upstream to the translation start codon, as previously inferred [33].

**Figure 6:**
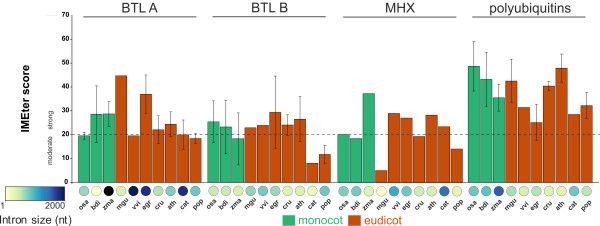
**IMEter score and 5′UTR intron size of *BTLs*, *MHX *and polyubiquitin genes.** IMEter scores and intron size for *BTL (A)* and *BTL (B)* genes are as in Figure 2, and for *MHX* and polyubiquitin genes enlisted in Additional file 1. Average values are considered on *BTL* and polyubiquitin genes, and the standard deviation is shown only for the IMEter score values. The intron size scale goes from 1 to 2000 nucleotides, one average value is longer that 2000 nucleotides (*Zea mays BTL(A)*, 3654.5 nt). A line at IMEter score 20 is shown across all species.

The average intron size of *MHX*, polyubiquitin genes and BTLs B was similar (MHX, 608.7+/-260 nucleotides; polyubiquitin genes, 607.2+/-258 nucleotides; 578+/-444 nucleotides) (Figure 6 and Additional file 1), suggesting that about 600 nucleotides is a common size for a 5′UTR intron in angiosperms. The overall IMEter scores ranged from moderate to strong values in *MHX* and *BTL* introns (from 10 to more than 20, Figure 6) and from strong values in polyubiquitin genes (more than 20, Figure 6), suggesting that the level of IME effect across species is conserved throughout angiosperms.

## Conclusions

Many BTL genes have spliceosomal introns located in the 5′UTR. Our findings suggest that this 5′UTR intron arose early in BTL evolution, as it was detected in two lineages that included both monocot and eudicot species. The IMEter score predicted that 5′UTR spliceosomal introns in *BTL*s encode regulatory elements that enhance gene expression, and our results showed that IMEter evaluation functions across a wide range of plant species. Analysis of promoter-GUS fusions lines indicated that the 5′UTR introns in *AthBTL1* and *AthBTL4* are important for gene expression in vegetative and reproductive tissues. Indeed, the IMEter scores of the two tested promoters were high, predicting that they may enhance gene expression in a strong manner. Comparison of IMEter scores of BTLs with known gene families, indicated that the IMEter score is conserved in members of a gene family across angiosperms, and that specifically, the 5′UTR intron of polyubiquitin genes resulted in higher values. It would be interesting to compare the extent of gene expression enhancement along members of these gene families to correlate the overall IMEter score with gene expression.

The use of 5′UTR intron for enhancement of gene expression in this class of E3 ligases is likely an effective mechanism, as our analysis suggests it has been preserved in evolution. Although the sizes of the 5′UTR introns in *AthBTL1* and *AthBTL4* are different, the introns exert a comparable enhancement of gene expression and similar expression domains in transgenic lines. Approximately 15% of BTLs in groups A and B do not have predicted introns in the 5′UTR. It would be interesting to evaluate whether these intronless sequences are expressed or whether the loss of the intron completely abolished gene expression, rendering these genes as pseudogenes.

## Methods

### Identification of 5′UTR introns in BTL, MHX and polyubiquitin genes from angiosperms

The peptide and predicted spliceosomal intron sequences were retrieved from fourteen angiosperm genomes deposited in the Phytozome 9.1 database at http://www.phytozome.net/. The genomes included four monocots and ten eudicot plants. If more than one spliceosomal intron was predicted in the 5′UTR, the largest intron sequence was considered. Only BTLs belonging to the previously identified groups A and B were studied. Spliceosomal intron sequences from the 5′UTR of *MHX* and polyubiquitin genes were retrieved from three monocot and seven eudicot species. In the polyubiquitin genes, the position of the intron was found immediately upstream to the ATG codon, except in three *Eucalyptus grandis* sequences, where the first intron within the coding DNA sequence was considered in the analysis.

### Phylogenetic analysis

Multiple alignment of the 81 retrieved BTL sequences was obtained by Clustal X [34]. The phylogenetic tree was based on concatenated RING-H2 and the BZF domains, and was generated using the Neighbor-Joining method with a Bootstrap value of 1000 replicates. The tree phylogeny was displayed on NJplot [35].

### IMEter scores

IMEter version 2.0 was used to obtain the IMEter scores: http://korflab.ucdavis.edu/cgi-bin/web-imeter2.pl. The transcribed strand was used in the analysis. A window of 80 nucleotides was used when the complete sequence from *A. thaliana* genes were scored. IMEter v2.0 scores were considered as: moderate enhancement (mod), between 10 and 20, and strong enhancement, more than 20.

### Generation of sequence LOGOs

Sequence LOGOs of donor and acceptor sites were obtained using MEME (Multiple EM for Motif Elicitation) version 4.9.0 at http://meme.nbcr.net/meme/cgi-bin/meme.cgi. Forty nucleotides spanning the donor or the acceptor sites were included in the sequence LOGO. The following parameters were used for analysis: zero or one per sequence, 40 amino acids as minimum and maximum sizes of motifs.

### Analysis of AthBTL1 and AthBTL4 expression in transgenic A. thaliana lines

*A. thaliana* ecotype Columbia (Col-0) was used in the floral dip transformation procedure with *Agrobacterium tumefaciens* strain GV2260. Transgenic plants were selected on MS agar medium containing 50 μg/ml kanamycin (Km). Plants were grown on MS agar medium or on soil under controlled environmental conditions at 16 h light/8 h dark cycles. A segregation test was performed by growing the seedlings from 25 independent lines of T2 generation under selective conditions. T2 lines that did not show segregation were eliminated from the analysis. Homozygous plants of the third generation (T3) were used for GUS staining experiments.

To obtain p*AthBTL1*: GUS and p*AthBTL4*: GUS transcriptional fusions, 2500 and 2200 bp fragments, respectively, were PCR amplified from genomic *A. thaliana* DNA and cloned into the vector pBI101 [36]. The reverse primer was designed to bind several nucleotides upstream of the assumed translation start codon. Oligonucleotides pairs used for molecular cloning of the promoter regions were as follows: pAthBTL1, 5′-TACTCGAGAGGAGGGGTCGTGTTAGTTG-3′ and 5′-TAGGATCCCAATGCAATGCTTTCCTTGA-3′; pAthBTL4, 5′-TAGTCGACGCAGTCAATAAGCGCAAGGT-3′ and 5′- ATGGATCCTTCCTTACTTCACCCCCACA-3′. The reverse oligonucleotides used for molecular cloning of the promoter regions without the intron sequences were as follows: for pAthBTL1 5′-TAGGATCCTAAGTCAACCCTACGTCTGC-3′, and for pAthBTL4 5′- ATGGATCCAAAATCTGTCCTTGCTTCTT-3′. Restriction site sequences were added at the 5′ and 3′ end of the primers to facilitate directional cloning into the pBI101.1 vector. The constructs were confirmed by sequencing.

GUS histochemical analysis was performed as previously described [36]. Tissues were analyzed under a Leica MZ12 stereomicroscope. Approximately 20 independent T2 segregating lines were initially stained for each of the constructs. For each construct, at least five lines that were followed until the T3 generation were used for the spatial-temporal expression analysis.

## Competing interests

The authors declare that they have no competing interests.

## Authors’ contributions

VA-H and PG conceived the study, VA-H performed all the analysis, PG wrote the manuscript. Both authors read and approved the final manuscript.

## Supplementary Material

Additional file 1 IMEter score and 5′UTR intron size of selected MHX and polyubiquitin genes. Click here for file
