# Supplementary material for: Spliceosomal introns in the 5′ untranslated region of plant BTL RING-H2 ubiquitin ligases are evolutionary conserved and required for gene expression
Source: BMC Plant Biol. 2013 Nov 14;13:179. doi: 10.1186/1471-2229-13-179 (PMC4225707; doi:10.1186/1471-2229-13-179)
Supplement: Additional file 1 — IMEter score and 5′UTR intron size of selected MHX and polyubiquitin genes. [file 1471-2229-13-179-S1.pdf]

Additional file 1:

IMEter score and 5'UTR intron size of selected *MHX* and polyubiquitin genes

	Species	Gene name	IMEter	Intron size
MHX genes	*Arabidopsis thaliana*	ath|At2g47600	27.42	416
	Aquilegia caerulea	aco|Aquca_003_00711	7.33	400
	Brachypodium distachyon	bdi|Bradi4g11030	17.92	816
	Cucumis sativus	cat|Cucsa.097070	22.71	1461
	Capsella rubella	cru|Carubv10022958m.g	18.75	448
	Eucalyptus grandis	egr|Eucgr.K03488	26.22	848
	Mimulus guttatus	mgu|mgv1a004377m.g	4.96	87
	Oryza sativa	osa|LOC_Os11g43860	19.58	777
	Populus trichocarpa	pop|POPTR_0014s12330	13.69	320
	Zea mays	zma|GRMZM2G126601	36.11	363
	Vitis vinifera	vvi|GSVIVG01028023001	28.09	1091
UBQ genes	*Arabidopsis thaliana*	ath|At4g05320/UBQ10	53.95	304
		ath|At5g20620/UBQ4	42	385
	Aquilegia caerulea	Aquca_005_00562	37.16	593
		Aquca_005_00454	37.39	812
	Brachypodium distachyon	Bradi1g32460	32.95	993
		Bradi1g32700	43.48	852
		Bradi1g32860	58.11	1090
		Bradi3g03957	25.83	450
		Bradi3g04730	44.6	959
		Bradi3g52300	55.1	1015
	Cucumis sativus	Cucsa.266970	28.59	828
	Capsella rubella	Carubv10001174m	40.39	324
		Carubv10001095m.g	38.16	405
		Carubv10020456m.g	40.22	411
		Carubv10001548m.g	42.25	381
		Carubv10001544m.g	41.53	392
	Eucalyptus grandis	Eucgr.F04448	33.66	807
		Eucgr.H03021	15.23	272
		Eucgr.K02023	26.7	407
	Mimulus guttatus	mgv1a004313m.g	31.67	406
		mgv1a003085m.g	52.67	609
		mgv1a004304m.g	35.64	744
		mgv1a004289m.g	50.46	698
	Oryza sativa	LOC_Os06g46770	43.95	698
		LOC_Os02g06640	63.22	962
		LOC_Os05g42424	39.22	885
	Populus trichocarpa	POPTR_0011s13770	34.28	584
		POPTR_0017s06450	39.98	560
		POPTR_0007s02460	33.48	612
		POPTR_0001s44440	22.97	550
		POPTR_0001s27020	30.62	532
	Zea mays	GRMZM2G419891	40.47	2386
		GRMZM2G409726	27.85	1010
		GRMZM2G118637	38.63	1329
	Vitis vinifera	GSVIVG01001971001	31.52	432
